# Magnesium deficit ? overlooked cause of low vitamin D status?

**DOI:** 10.1186/1741-7015-11-229

**Published:** 2013-10-24

**Authors:** Armin Zittermann

**Affiliations:** 1 Clinic for Thoracic and Cardiovascular Surgery, Heart Center North Rhine-Westphalia, Ruhr-University of Bochum, Georgstra?e 11, 32545, Bad Oeynhausen Germany

**Keywords:** 25-hydroxyvitamin D, Cardiovascular mortality, Magnesium, Mortality, Vitamin D deficit

## Abstract

Like vitamin D deficit, magnesium deficit is considered to be a risk factor for cardiovascular disease. Several steps in the vitamin D metabolism, such as vitamin D binding to its transport protein and the conversion of vitamin D into the hormonal form 1,25-dihydroxyvitamin D by hepatic and renal hydroxylation, depend on magnesium as a cofactor. A new analysis of two National Health and Nutrition Examination Surveys data sets, published in *BMC Medicine*, investigated potential interactions between magnesium intake, circulating 25-hydroxyvitamin D, which is the generally accepted indicator of vitamin D status, and mortality. Data indicate a reduced risk of insufficient/deficient vitamin D status at high magnesium intake and an inverse association between circulating 25-hydroxyvitamin D and mortality, particularly cardiovascular mortality, among those with magnesium intake above the median. The study provides important findings concerning potential metabolic interactions between magnesium and vitamin D and its clinical relevance. However, results should be considered preliminary since biochemical data on individual magnesium status were lacking, confounding cannot be excluded and questions on the dose?response relationship still remain to be answered.

Please see related research article: http://www.biomedcentral.com/1741-7015/11/187.

## Background

Life depends on an energy-consuming complex interplay of organic and inorganic substances to maintain biological structures. Adequate energy and nutrient supply is a prerequisite to guarantee normal functioning of metabolic pathways and thus a healthy life. To become metabolically active, several nutrients require other essential nutrients as cofactors. For example, copper is required for the oxidation of absorbed Fe^2+^ to Fe^3+^, which is then bound to transferrin, and riboflavin (vitamin B_2_) and pyridoxine (vitamin B_6_) are required to produce niacin (vitamin B_3_) from dietary tryptophan. Therefore, some nutrition-related illnesses, such as anemia and pellagra, can be caused by multiple nutrient deficits [1,2]. Magnesium (Mg) is a cofactor that is required for the binding of vitamin D to its transport protein. Moreover, conversion of vitamin D by hepatic 25-hydroxlation and renal 1?-hydroxylation into the active, hormonal form 1,25-dihydroxyvitamin D (1,25(OH)_2_D) is Mg-dependent [3,4]. A study by Deng *et al*. [5], published in *BMC Medicine*, investigated potential interactions between Mg intake, vitamin D status and mortality. Still, some questions remain open.

## Study results

Deng *et al*. [5] used two large National Health and Nutrition Examination Surveys data sets to assess interactions between Mg intake, vitamin D status and outcome. According to the Institute of Medicine (IOM) classification, circulating 25-hydroxyvitamin D (25OHD), the generally accepted indicator of vitamin D status, was within the deficit range (<12 ng/ml) in 12% of participants and the insufficiency range (12 to 20 ng/ml) [6] in 30%. Mean energy-adjusted total Mg intake (dietary and supplemental) was clearly below the recommended daily allowance, which is between 310 and 420 mg depending on age and gender [7]. High Mg intake was associated with reduced risk of vitamin D deficit or insufficiency. Data also indicate an inverse association between circulating 25OHD and mortality, particularly cardiovascular mortality, among those with Mg intake above the median.

## Vitamin D status and its predictors

The present data are consistent with the assumption that deficient vitamin D levels are an important health issue that may affect not only musculoskeletal health but also a wide range of acute and chronic diseases [8]. The high prevalence of deficient 25OHD blood levels in the population is believed to be primarily due to inadequate vitamin D synthesis in the skin, together with inadequate dietary intake [9]. However, circulating 25OHD levels also depend on body weight and genetic factors, and a significant portion of the variance in circulating 25OHD remains unexplained at present. Therefore, Deng *et al*. are to be commended for investigating potential interactions between Mg and vitamin D status. Especially with regard to vitamin D, there is still ongoing debate regarding the optimal oral intake and the adequate circulating 25OHD level. The IOM recommends a daily vitamin D intake of 600 international units and considers circulating 25OHD levels of 20 to 50 ng/ml as adequate, and 25OHD levels above 50 ng/ml as potentially harmful [6]. However, according to the Endocrine Society [10], the circulating 25OHD level should be within the range of 30 to 100 ng/ml. To raise the 25OHD level consistently above 30 ng/ml, they recommend a daily vitamin D intake of 1,500 to 2,000 international units.

## Open questions

Like vitamin D deficit [11], Mg deficit is considered to be a risk factor for cardiovascular disease [12]. Thus, the presented interactions between Mg, vitamin D and cardiovascular mortality fit well together. Nevertheless, for several reasons, data should be considered preliminary. First, associations are based on oral Mg intake. The assessment of oral Mg intake does not take into account inter-individual differences in intestinal Mg absorption and renal and cutaneous Mg loss. It would have been more appropriate to use biochemical data, such as plasma Mg levels (levels of 0.79 to 0.76 mmol/l have been classified as inadequate and levels <0.76 mmol/l as deficient), to assess individual Mg status. Unfortunately, plasma levels of Mg are still rarely assessed, whereas measurement of circulating 25OHD levels has exploded in the clinical setting in recent years [13]. Second, total Mg intake, 25OHD status and physical activity level were clearly interrelated [5]. High Mg intake may thus only be an indicator for high physical activity, which is known to influence 25OHD status (and cardiovascular mortality). Even adjustment for physical activity may not completely solve the problem of potential bias. Third, no data on circulating levels of the biologically active form of vitamin D, 1,25(OH)_2_D, were presented. Circulating 1,25(OH)_2_D is probably a better predictor of total mortality compared with circulating 25OHD, at least if mid-term mortality is assessed [14]. Circulating 1,25(OH)_2_D declines significantly (and parathyroid hormone levels increase substantially) at 25OHD levels below 10 ng/ml, whereas levels of both hormones seem to plateau at 25OHD levels of 20 ng/ml [15]. Notably, three out of the four 25OHD categories in the mortality analysis of the present investigation were above 20 ng/ml, and none covered the range that the IOM has classified as deficient. It would have been interesting to see whether the combination of inadequate Mg intake with 25OHD levels below 12 ng/ml is associated with a further mortality increase.

## Conclusion

The present study must be praised for bringing potential interactions between individual nutrients into the focus of interest. Because the intake of Mg is often inadequate and several other factors are also known to impair Mg supply (for example, diuretics use, diabetes mellitus, chronic alcohol consumption, stress factors) [16-18], more attention should in future be paid to possible consequences of insufficient or deficient Mg supply in the general population. Further studies on the interactions between Mg supply and vitamin D status should include a more detailed assessment of individual Mg status (for example, by measuring biochemical parameters of Mg status or by adjusting statistical analysis for stress factors, diuretics use, alcohol consumption and concomitant diagnoses such as diabetes); a more detailed investigation of different components of the vitamin D-parathyroid hormone axis in general populations; clarification of the dose?response relationship; and the realization of randomized controlled trials to verify whether oral Mg is indeed able to improve vitamin D status and survival.

## Abbreviations

1,25(OH)2D: 1,25-dihydroxyvitamin D; 25OHD: 25-hydroxyvitamin D; IOM: Institute of Medicine; Mg: Magnesium.

## Competing interests

The author declares that he has received speaker honoraria from Abbott, Germany, and DiaSorin, Germany, two companies that provide test kits for the measurement of circulating 25-hydroxyvitamin D.

## Author information

AZ is a nutritionist, whose main research focuses on human vitamin D status, including the prevalence and consequences of vitamin D insufficiency or deficit. He is head of the study center at the Clinic for Thoracic and Cardiovascular Surgery at the Heart Center North-Rhine Westphalia, Ruhr-University of Bochum, Germany.
